# Hypoglycemic Effect of the N-Butanol Fraction of *Torreya grandis* Leaves on Type 2 Diabetes Mellitus in Rats through the Amelioration of Oxidative Stress and Enhancement of *β*-Cell Function

**DOI:** 10.1155/2022/5648896

**Published:** 2022-12-28

**Authors:** Xue-Qin Li, Shan-Shan Jia, Ke Yuan, Song-Heng Jin

**Affiliations:** Jiyang College of Zhejiang A and F University, Zhu'ji 311800, China

## Abstract

**Materials and Methods:**

Sprague–Dawley rats were randomly divided into six groups: control, T2DM, metformin, high-dose BFTL (800 mg/kg), middle-dose BFTL (400 mg/kg), and low-dose BFTL (200 mg/kg). After 4 weeks of BFTL treatment, the correlations of serum indicators with protein expression in tissue were determined, and pathological changes in the liver, kidneys, and pancreas were analyzed.

**Results:**

Compared with the results in the T2DM group, serum fasting blood glucose, triglyceride, total cholesterol, malondialdehyde, alanine aminotransferase, and aspartate aminotransferase levels were significantly decreased (*p* < 0.05), whereas superoxide dismutase and glutathione peroxidase levels were significantly increased (*p* < 0.05) in the high-, middle-, and low-dose BFTL groups. The treatment also improved oral glucose tolerance. In addition, the pathological changes of the liver, kidney, and pancreas were improved by BFTL treatment. Cytochrome and caspase-3 expression in pancreatic was significantly decreased (*p* < 0.05) by BFTL treatment, whereas the Bcl-2/Bax ratio was significantly increased (*p* < 0.05). *Discussion and Conclusion*. BFTL exerted significant hypoglycemic effect on T2DM model rats, and its mechanism involved the suppression of blood glucose levels and oxidative stress by improving the metabolism of blood lipids and antioxidant capacity, boosting *β*-cell function, and inhibiting *β*-cell apoptosis.

## 1. Introduction

Diabetes is a common clinical chronic disease caused by endocrine and metabolic disorder [1, 2]. The main symptom of type 2 diabetes mellitus (T2DM) is insulin (INS) resistance accompanied by a relative or absolute insufficiency of INS secretion, which leads to metabolic disorders of sugar, fat, and protein. The clinical manifestations of T2DM include long-term continuous hyperglycemia, dehydration, increased hunger, high urinary frequency, and weight loss, with many complications resulting in high mortality [3–5]. Diabetes is often accompanied by neuropathy, nephropathy, retinopathy, cardiovascular and cerebrovascular diseases, and a series of diabetic complications [6, 7].

For a long time, western medicine has been mainly adopted for the treatment of diabetes. The commonly used drugs in clinical practice mainly include INS and metformin (Met). However, the toxic and side effects of these drugs should not be underestimated [8–10]. The active ingredients of plants have attracted increasing attention from researchers because of their high efficacy and low side effects [11]. In a study on adult diabetic rats, it was shown that treatment with carvacrol reduced the tissue activity of superoxide dismutase (SOD) and glutathione peroxidase (GPx) enzymes, and diminished the elevated levels of tissue malondialdehyde (MDA) [12, 13]. Study showed that hydroalcoholic extracts of cloves (Syzygium aromaticum) had beneficial effects in diabetes through improving glycemic control and lipid profile and preventing from diabetes-induced kidney damages [14]. Another study also came to the same conclusion in antidiabetic effects of *Galega officinalis* (an herbal medicine) on diabetic rats [15]. Therefore, the isolation of ideal active substances from plants to prevent and treat diabetes and clarification of its complications are the main trends of modern medical research.

*Torreya grandis* (*T. granids*), also known as *Torreya chinensis*, is an evergreen tree [16]. The plant material was identified by Professor Pin-Zhang Tong, the plant taxonomy expert of Forestry Bureau Zhuji, Zhejiang Province. It is one of the rare economic species of dried fruit in China, and it is mainly distributed in Anhui, Jiangsu, Zhejiang, and other provinces south of the Yangtze River [17]. It has been reported that *T. grandis* leaves mainly contain volatile oils, terpenoids, flavonoids, alkaloids, tannins, and saponins [18]. The water and ethanol extracts of *T. grandis* leaf have different degrees of analgesic, anti-inflammatory, hypolipidemic, hypocholesterolemic, antioxidant, antibacterial, and immunomodulatory effects [19, 20]. Currently, few studies have examined *T. grandis* leaves. This paper studied the hypoglycemic effects of the n-butanol fraction of the 75% ethanol extract of *T. grandis* leaves (BFTL) on T2DM model rats and explored its potential mechanism regarding lipid metabolism, oxidative stress, and cell apoptosis to provide a reference for the further development and utilization of *T. grandis* leaf resources.

## 2. Material and Methods

### 2.1. Materials and Reagent

Infinite M200 enzyme marker (Tecan, Switzerland); UV-2102 PCS spectrophotometer (Unico Instruments Co., Lt., Shanghai); BX20 optical microscope (Tokyo, Japan); flash distillation concentration device (self-designed) [21], KQ-250B ultrasonic cleaner (Kunshan Ultrasonic Instruments Co., Ltd., Kunshan, China); IKA RV8 V rotary evaporator (Shanghai Shensheng Biotechnology Co., Ltd., Shanghai, China); LG10-2.4A high-speed centrifuge (Beijing Medical Centrifuge Factory, Beijing, China); All other reagents are analytical pure reagents.

*Torreya grandis* (*T. granids*) leaf was obtained from Zhuji, Zhejiang Province, China in September 2014. Streptozocin (STZ) was purchased from Aladdin Reagent Company (China, Shanghai); Metformin (Met) was purchased from Yunnan Kunming Pharmaceutical Co., Ltd. (China, Kunming); fasting blood glucose, insulin, superoxide dismutase (SOD), malondialdehyde (MDA), glutathione peroxidase (GSH-PX), triglyceride (TG), total cholesterol (TC), alanine aminotransferase (ALT), and aspartate aminotransferase (AST) kits were purchased from Nanjing Jiancheng Biotechnology Co., Ltd. (China, Nanjing). Cyt-c, caspase-3, Bcl-2, and Bax antibodies were purchased from Xinbosheng Technology Co., Ltd. (Shanghai, China); Chloral hydrate was purchased from Qingdao Yulong Seaweed Co., Ltd. (Qingdao, China).

### 2.2. Preparation of BFTL

Dried and crushed *T. grandis* leaves were immersed in cold 75% ethanol for 12 h and then extracted at room temperature. The aforementioned procedures were repeated three times, and the extracted solutions were combined and concentrated by a vacuum thin-film concentration device at 60°C [22]. The concentrated solution was dispersed by ultrasound in an appropriate amount of water and then extracted successively with petroleum ether, ethyl acetate, and n-butanol. Vacuum concentration was conducted using a rotary evaporator to obtain a dry powder of the different extraction fractions. Among them, the dried powder yield of the n-butanol fraction of the ethanol extract was 356 g.

### 2.3. Design of Animal Experiments

Four-week-old male Sprague–Dawley rats (190 ± 10 g) were purchased from the Experimental Animal Center of Zhejiang Academy of Medical Sciences (Zhejiang, China). The production license number of experimental animals was SCXK 2015-0033. All experimental animals were kept in a standard animal house with an ambient temperature of 23 ± 1°C and relative humidity of 50 ± 5% for 1 week before the experiment [23], after which they were then randomly divided into six groups (*n* = 10): normal control (NC), T2DM, Met (100 mg/kg), low-dose BFTL (200 mg/kg), middle-dose BFTL (400 mg/kg), and high-dose BFTL (800 mg/kg). Excluding rats in the NC group, which were fed normal food (10% water, 18% protein, 4% fat, 5% fiber, 8% ash content, 1% calcium, and 1% phosphorus) and water, the experimental rats in the other groups were fed a high-fat diet (10% water, 18% protein, 24% fat, 5% fiber, 8% ash content, 2.5% cholesterol, 1% calcium, and 1% phosphorus) and high-sucrose water (containing 20% sucrose) [24] for 4 weeks and then injected intraperitoneally with streptozotocin (STZ, 30 mg/kg) three times every other day [25]. Fasting blood glucose (FBG) content was measured 72 h after the last STZ dose. An FBG level exceeding 11.1 mol/L indicated that diabetes was successfully established [26]. Next, the rats that had been successfully modeled were intraperitoneally injected once a day with normal saline (NC and T2DM groups), Met, or the indicated dose of BFTL. During this period, body weight and the amounts of food and water intake were recorded daily. Treatment was continued for 4 weeks. Rat orbital blood was taken, and FBG levels were measured once a week. After the last dose of BFTL, rats were fasted for 12 h and anesthetized with ketamine 80 mg/kg by intraperitoneal injection. Blood was taken from the abdominal aorta, and the serum was centrifuged and separated. The liver, kidneys, and pancreas were removed and then stored at −80°C for further study. All experimental procedures were performed in accordance with the Guide for the Care and Use of Laboratory Animals of Zhejiang and approved by the Committee on the Ethics of Animal Experiments at Animal Center of Zhejiang Agriculture and Forestry University.

### 2.4. Determination of Serum Biochemical Indices in Rats

#### 2.4.1. Determination of Body Weight, FBG, and Oral Glucose Tolerance Serum

During the experiment, the weight, food intake, and water intake of the rats were measured, and the clinical symptoms of ‘three more and one less' of diabetes, namely eating more, drinking more, urinating more, and losing weight, were examined, as was symptom relief after treatment. Blood was collected from the tail vein, and serum was separated. Then, 2000 mg/kg glucose solution was given, and blood samples were taken from the vein of the tail at 30, 60, and 120 min [27]. The FBG content and oral glucose tolerance were measured according to the instructions of the enzyme-linked immunoassay kit.

#### 2.4.2. Determination of INS, Triglyceride (TG), Total Cholesterol (TC), Malondialdehyde (MDA), Superoxide Dismutase (SOD), Glutathione Peroxidase (GSH-PX), Alanine Aminotransferase (ALT), and Aspartate Aminotransferase (AST) Levels in Serum

INS, TG, TC, MDA, SOD, GSH-PX, ALT, and AST levels in serum were determined according to the kit instructions.

### 2.5. Determination of Liver and Kidney Indices in Rats

The liver and bilateral kidneys of rats were, respectively, removed and weighed, and liver and kidney indices were calculated.

### 2.6. Histopathology

Liver, kidney, and pancreatic tissues were fixed in 4% neutral formaldehyde for 24 h and then embedded in paraffin. Sections of 5 *μ*m were cut and stained with hematoxylin and eosin (H&E) [28], and the pathological changes of the tissues were observed under an optical microscope.

### 2.7. Western Blotting (WB) Analysis of Protein Expression in Pancreas Tissue

After obtaining pancreatic tissues, protein lysis buffer was added, tissues were homogenized, the supernatant was collected after centrifugation, and the protein content was determined by the Bicinchoninic acid (BCA) method [29]. The protein was separated by electrophoresis on a polyacrylamide gel, electrically transferred to a PVDF cellulose membrane, and sealed with a saline solution containing 50 g/L skim milk powder at room temperature for 1 h. Then, membranes were incubated with antibodies against cytochrome c (Cyt-c), caspase-3, Bcl-2, and Bax were incubated at 4°C overnight and washed with TBST. Peroxidase-labeled sheep anti-rabbit secondary antibody was incubated at room temperature for 1 h. After three washes with TBST, an ECL reagent was added, and images were captured using a chemiluminescence detection system. *β*-Actin was used as the internal reference to determine protein expression.

### 2.8. Statistical Analysis

Data were expressed in the form of mean ± standard deviation. SPSS 19.0 (Inc., Chicago, IL, USA) was using to statistical. One-way analysis of variance (ANOVA) was used. *p* < 0.05 was considered a significant difference, and *p* < 0.01 was considered an extremely significant difference.

## 3. Results and Discussion

### 3.1. Changes In Body Weight, Organ Indices, and Blood Glucose Levels

**Figure 1:**
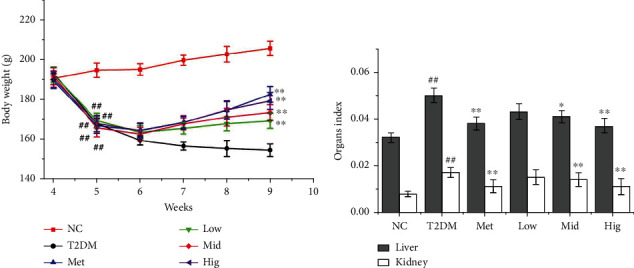
Effect of BFTL on body weight (a) and index of liver and kidney (b) in rats. NC: negative control; T2DM: type 2 diabetes mellitus; Met: metformin; Low: BFTL (200 mg/kg); Mid: BFTL (400 mg/kg); Hig: BFTL (800 mg/kg). The data were expressed as mean ± SD (*n* = 10), #*p* < 0.05, ##*p* < 0.01 vs. NC group; ^∗^*p* < 0.05, ^∗∗^*p* < 0.01 vs. T2DM group.

‘Three more and one less' denotes the main clinical features of diabetes. As body mass decreases, organ mass changes accordingly. As presented in Figure 1(a), starting from the moment STZ was injected in week 4–5, body mass was significantly lower in the T2DM group than in the NC group (*p* < 0.01). Meanwhile, body mass was significantly increased in the Met and BFTL groups compared with that in the T2DM group (*p* < 0.01), and body mass gradually increased with prolonged treatment time, indicating that BFTL can significantly increase the body mass of rats. As presented in Figure 1(b), the liver and kidney indices rats were higher significantly in the T2DM group than in the NC group (*p* < 0.01), whereas the indices were significantly lower in the middle- and high-dose BFTL and Met groups than in the T2DM group (*P* < 0.05). The effect on liver and kidney indices was strongest in the high-dose BFTL groups (*p* < 0.01). These results illustrated that diabetes significantly decreased the weight of mice, whereas BFTL treatment reversed the changes of body weight and liver and kidney indices associated with diabetes.

**Figure 2:**
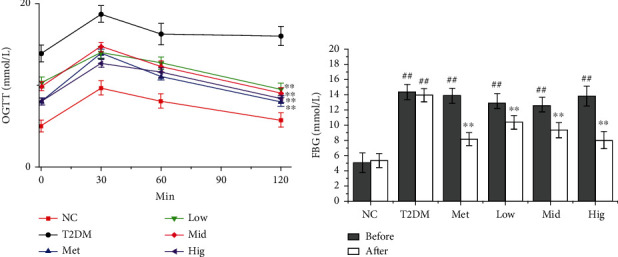
Effect of BFTL on OGTT (a) and FBG (b) of rats. NC: negative control; T2DM: type 2 diabetes mellitus; Met: metformin; Low: BFTL (200 mg/kg); Mid: BFTL (400 mg/kg); Hig: BFTL (800 mg/kg). The data were expressed as mean ± SD (*n* = 10), #*p* < 0.05, ##*p* < 0.01 vs. NC group; ^∗^*p* < 0.05, ^∗∗^*p* < 0.01 vs. T2DM group.

As presented in Figure 2(a), oral glucose tolerance was lower in the T2DM group than in the NC group at 0, 30, 60, and 120 min (*p* < 0.01), indicating that INS resistance had developed. Compared with the results in the T2DM group, oral glucose tolerance was significantly improved in all three BFTL groups at 0, 30, 60, and 120 min versus the findings in the T2DM group (*p* < 0.05). Elevation of blood glucose levels is the main clinical feature of diabetes. As presented in Figure 2(b), FBG levels were significantly higher in the T2DM group than in the NC group (*p* < 0.01). Compared with the results in the T2DM group, FBG levels were significantly decreased by Met or BFTL treatment (*p* < 0.01). The results indicated that BFTL could significantly improve oral glucose tolerance in diabetic rats, and the effects were more obvious in the high-dose BFTL groups. A similar result was showed FBG levels were significantly reduced in type 2 diabetic rats after treatment ([14]; Wei et al. 2017).

### 3.2. Effects of BFTL on Serum Indices

**Figure 3:**
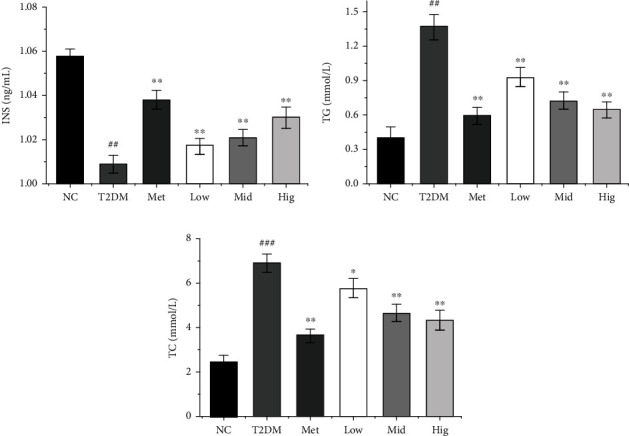
Effect of BFTL on INS (a), TG (b), and TC (c) of rats. NC: negative control; T2DM: type 2 diabetes mellitus; Met: metformin; Low: BFTL (200 mg/kg); Mid: BFTL (400 mg/kg); Hig: BFTL (800 mg/kg). The data were expressed as mean ± SD (*n* = 10), #*p* < 0.05, ##*p* < 0.01 vs. NC group; ^∗^*p* < 0.05, ^∗∗^*p* < 0.01 vs. T2DM group.

INS is secreted by islet *β*-cells and is mainly used to evaluate the function of islet *β*-cells. When T2DM occurs, the relative or absolute insufficiency of INS secretion will cause a series of metabolic disorders [30, 31]. As presented in Figure 3(a), serum INS content was significantly lower in the T2DM group than in the NC group (*p* < 0.01), whereas INS levels were significantly higher in the BFTL groups (*p* < 0.01) with a certain dose dependence. This demonstrated that BFTL can significantly improve INS content.

Disordered lipid metabolism is an important characteristic of T2DM that can lead to an abnormal increase in the content of various lipid components in the blood [32]. Large amounts of glucose and free fatty acids (FFAs) enter the liver and lead to increases of TC and TG levels, resulting in INS resistance [33]. When TG levels remain elevated levels, heparin activates lipoprotein lipase and increases the intravascular lipolysis of TG, thereby increasing tissue exposure to FFAs and leading to INS resistance and *β*-cell functional damage [34]. TC represents the sum of cholesterol contained in all lipoproteins in blood, and it is closely related to various complications such as diabetes, cardiovascular and cerebrovascular diseases, and neuropathy [35]. Therefore, improving the body's lipid metabolism can improve diabetes to a certain extent [36]. As presented in Figures 3(b) and 3(c), serum TG and TC levels were significantly higher in the T2DM group than in the NC group (*p* < 0.01). Meanwhile, serum TC and TG levels were significantly decreased after 4 weeks of Met or BFTL administration (*p* < 0.05) versus the findings in the T2DM group, especially in the middle- and high-dose groups. This indicates that BFTL can effectively improve serum TG and TC levels in diabetic rats, thereby inhibiting lipid peroxidation. Similarly, the study of Wei et al. (Wei et al. 2020) showed that T2DM rats had higher levels of TC, total triglyceride (TG) compared with normal, healthy SD rats in Tanshinone I alleviates insulin resistance in type 2 diabetes mellitus rats.

**Figure 4:**
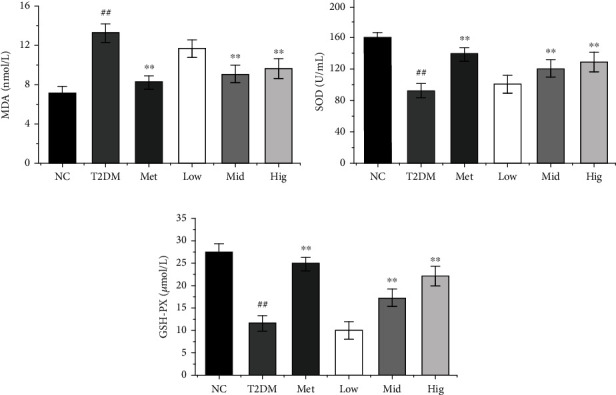
Effect of BFTL on MDA (a), SOD (b), and GSH-PX (c) of rats. NC: negative control; T2DM: type 2 diabetes mellitus; Met: metformin; Low: BFTL (200 mg/kg); Mid: BFTL (400 mg/kg); Hig: BFTL (800 mg/kg). The data were expressed as mean ± SD (*n* = 10), #*p* < 0.05, ##*p* < 0.01 vs. NC group; ^∗^*p* < 0.05, ^∗∗^*p* < 0.01 vs. T2DM group.

Oxidative stress is an imbalance between oxidative and antioxidant reactions in the body, which leads to excessive production of oxygen free radicals, which will create a peroxidative environment in the body, and this environment will disrupt the cell repair process and lead to cell damage. Meanwhile, oxidative stress is an important causative factor in chronic diseases such as type 2 diabetes. The antioxidant system scavenges oxygen radicals and the degree of oxidative damage in the body can be responded by the activity of enzymatic antioxidants such as CAT, SOD, and GSH-Px [37]. A large number of studies have revealed that oxidative stress is closely related to the occurrence of diabetes. Hyperglycemia can increase oxidative stress and the production of lipid peroxidation, and oxidative stress can regulate the secretion of INS in different ways and accelerate the development of diabetes [38]. Increased oxidative stress can promote *β*-cell dysfunction or apoptosis through INS resistance, which further inhibits INS secretion [36]. SOD, GSH-PX, and MDA in serum are important substances in the process of oxidative stress that reflect the body's antioxidant capacity [39]. As presented in Figures 4(a)–4(c), serum SOD and GSH-PX levels were significantly decreased and MDA levels were significantly increased by the development of diabetes versus the findings in the NC group (*p* < 0.01). Compared with the results in the T2DM group, serum SOD and GSH-PX levels were increased after 4 weeks of Met administration and significantly enhanced by BFTL treatment in a dose-dependent manner (*p* < 0.01), whereas serum MDA levels were reduced by Met or BFTL treatment (*p* < 0.01), highlighting that middle- and high-dose BFTL can improve the metabolism of sugar and lipids in rats with diabetes, reduce the body's levels of oxidative stress, reverse the dysfunction of INS-producing *β*-cells, and promote INS secretion. Wan et al. [40] showed that the silkworm extract treatment significantly increased SOD levels in diabetic rats, while decreasing MDA levels. So, our results indicated that BFTL treatment may treat diabetes by relieving oxidative stress.

**Figure 5:**
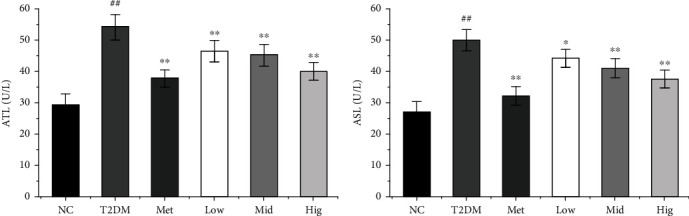
Effect of BFTL on ALT (a) and AST (b) of rats. NC: negative control; T2DM: type 2 diabetes mellitus; Met: metformin; Low: BFTL (200 mg/kg); Mid: BFTL (400 mg/kg); Hig: BFTL (800 mg/kg). The data were expressed as mean ± SD (*n* = 10), #*p* < 0.05, ##*p* < 0.01 vs. NC group; ^∗^*p* < 0.05, ^∗∗^*p* < 0.01 vs. T2DM group.

The liver is an important organ for regulating blood glucose. The persistent high blood glucose and long-term metabolic disorder of diabetes can lead to systemic organ damage and dysfunction, especially liver, kidney, and cardiovascular damage [41, 42]. Liver injury is one of the common complications of diabetes that leads to the elevation of ALT and AST levels [43]. AST is mainly distributed in the myocardium, followed by the liver, skeletal muscle, kidneys, and other tissues. Under normal conditions, AST content in the serum is low. However, when the corresponding cells are damaged, the permeability of the cell membrane will increase, and AST in the cytoplasm is released into the blood, leading to increased serum AST concentrations [44]. So, we detected the levels of ALT and AST in serum. As presented in Figures 5(a) and 5(b), serum ALT and AST levels were significantly higher in the T2DM group than in the NC group (*p* < 0.01). Compared with the results in the T2DM group, serum ALT and AST levels were significantly reduced after 4 weeks of administration in the Met and BFTL groups (*p* < 0.05), indicating that BFTL could effectively reduce ALT and AST levels in the serum of diabetic rats and high-dose BFTL had the strongest effect. Among all our serum biochemical indicators showed that BFTL had the potential hypoglycemic effect.

### 3.3. Effects of BFTL on Histopathology

**Figure 6:**
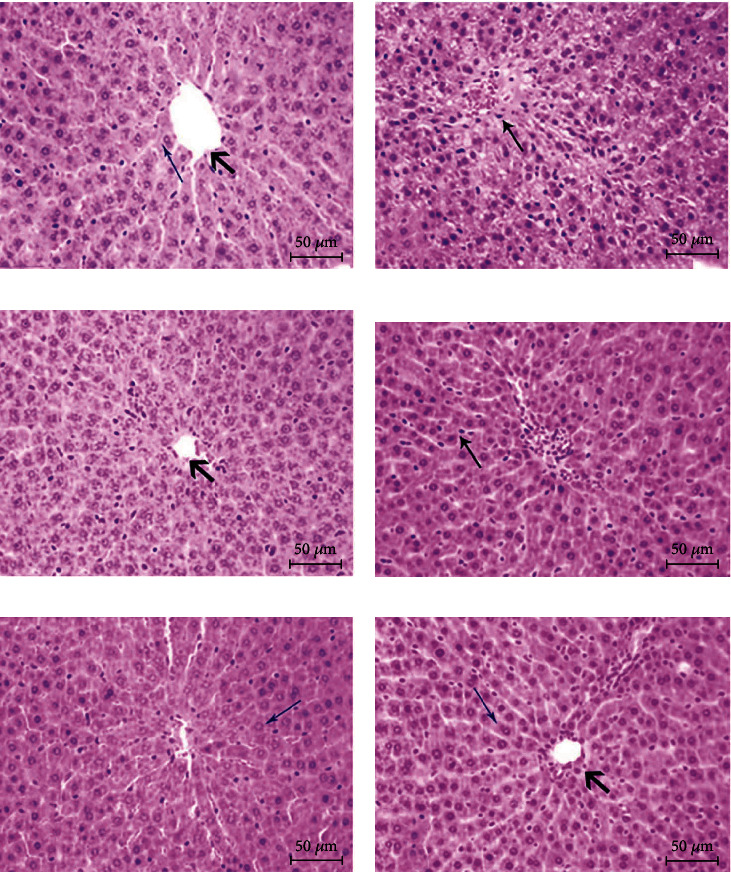
Effect of BFTL on liver pathological of rats. Histological observation, H&E, (a–f) ×200; (a) NC group; (b) T2DM group; (c) Met group; (d) BFTL (200 mg/kg); (e) BFTL (400 mg/kg); (f) BFTL (800 mg/kg); (⟶) central veins; (⟶) hepatocyte; (⟶) inflammatory cell.

Hyperglycemia both affects the transmission of islet cell signals and damages major tissues and organs [45]. As one of the targets of the main metabolic organs, the liver is the main site of injury caused by diabetes.  Figure 6 shows the hematoxylin and eosin (H&E) staining of liver tissue. The liver tissue structure in the NC group was complete and clear, and the cytoplasm of cells was clear. Hepatocytes were arranged radially around the central vein in a cord-like manner. Compared with the findings in the NC group, the liver cords in the T2DM group were disordered and irregular, and the liver cells had obvious infiltration and degeneration of inflammatory cells. The T2DM group perfectly demonstrated the specific manifestations of liver injury, such as steatosis, inflammatory cell infiltration, and cell necrosis [46]. Relative to the T2DM group, liver tissue lesions in the Met and BFTL groups were improved by different degrees. The number of inflammatory cells was significantly reduced, and the hepatocytes were neatly arranged.

**Figure 7:**
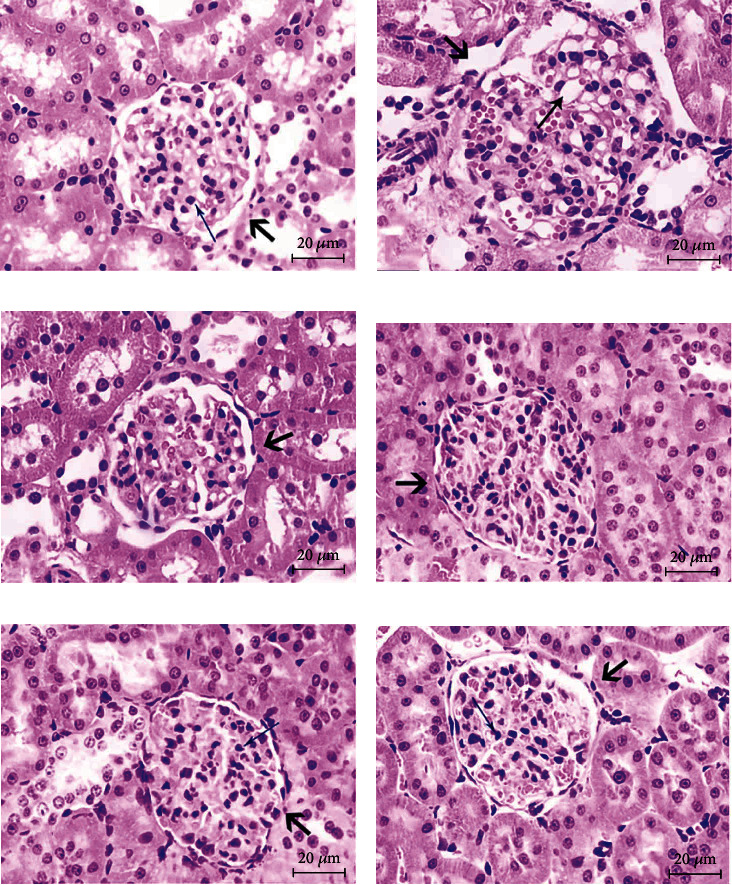
Effect of BFTL on kidney pathological of rats. Histological observation, H&E, (a–f) ×400; (a) NC group; (b) T2DM group; (c) Met group; (d) BFTL (200 mg/kg); (e) BFTL (400 mg/kg); (f) BFTL (800 mg/kg); (⟶) glomerulus; (⟶) vacuole; (⟶) interstitial cell.

Decreases of SOD and GSH-PX levels and increases of lipid peroxide and free radical levels promote the expression of transforming growth factor (TGF-*β*1) in renal tissue cells *in vivo*. TGF-*β*1 can promote renal cell hypertrophy, leading to glomerulosclerosis and renal interstitial fibrosis [47]. Therefore, the kidney tissue was evaluated with hematoxylin and eosin (H&E) staining. As presented in Figure 7, the glomeruli of rats in the NC group had no vacuoles, the renal tubules were normal, and the renal tissue structure was clear. Compared with the results in the NC group, rats in the T2DM group had dilated renal tubules, an increased number of glomerular cells, an increased renal volume, and a disordered renal structure. Compared with the results in the T2DM group, the rats in the Met and BFTL groups displayed significant improvements in the glomeruli, renal tubules, and other pathological tissues.

**Figure 8:**
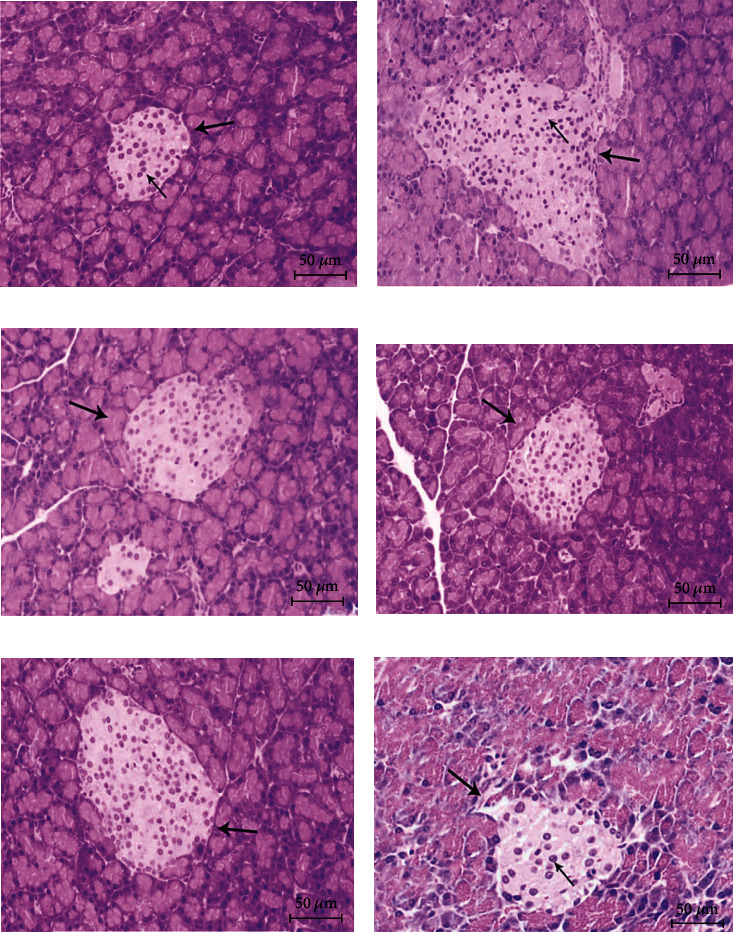
Effect of BFTL on pancreas pathological of rats. Histological observation, H&E, (a–f) ×200; (a) NC group; (b) T2DM group; (c) Met group; (d) BFTL (200 mg/kg); (e) BFTL (400 mg/kg); (f) BFTL (800 mg/kg); (⟶) islet; (⟶) islet cell.

Patients with T2DM have persistent hyperglycemia and hyperlipidemia. Long-term exposure to high concentrations of sugar or lipids will both cause functional dysfunction of islet cells and damage the tissue structure of islets [48]. As presented in Figure 8, the islet shape of rats in the NC group was regular, and the cells in the islet were arranged neatly. Compared with NC rats, the shape of the islet in rats in the T2DM group was obviously changed, and the boundary was blurred. Compared with T2DM rats, the histopathology of the islets of rats in the Met and BFTL groups exhibited varying degrees of improvement, and the improvement was dose-dependent.

## 4. Effects of BFTL on the Expression of Cyt-c, Caspase-3, Bcl-2, and Bax in Pancreatic Tissue

Sustained hyperglycemia can lead to excessive free radical production through the spontaneous oxidation of glucose. Therefore, diabetes is often manifested as an abnormal increase of reactive oxygen species (ROS) [49]. An abnormally high ROS level can impair the function of endoplasmic reticula and mitochondria, thereby damaging cells and leading to apoptosis. The caspase and Bcl-2 families play important roles in the cascade reaction of apoptosis. Bcl-2 is an antiapoptotic protein, whereas Bax is a proapoptotic protein. Their ratio regulates the membrane potential of mitochondria [20]. Bcl-2 downregulation and Bax upregulation can reduce mitochondrial membrane potential, thus inducing Cyt-c release and apoptotic body formation. Apoptotic bodies can also activate procaspase-9, which can lead to the cascade reaction of caspase-9 and activation of downstream caspase-3, ultimately leading to apoptosis [50, 51].

**Figure 9:**
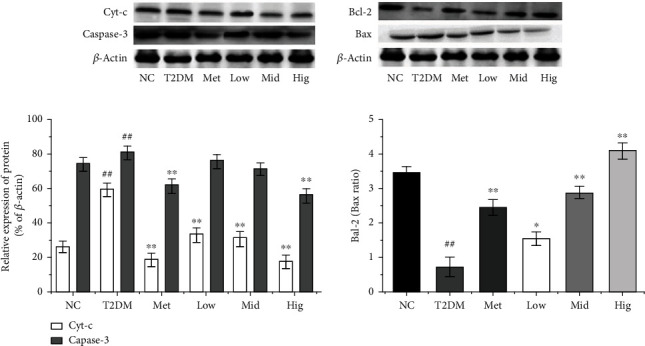
Effect of BFTL on the expression level of Cyt-c, caspase-3, Bcl-2, and Bax proteins. (a) The expression levels of Cyt-c, caspase-3, Bcl-2, and Bax proteins were analyzed by Western blotting. (b) Quantitative results of the expression levels. NC: negative control; T2DM: type 2 diabetes mellitus; Met: metformin; Low: BFTL (200 mg/kg); Mid: BFTL (400 mg/kg); High: BFTL (800 mg/kg). The data were expressed as mean ± SD (*n* = 10), #*p* < 0.05, ##*p* < 0.01 vs. NC group; ^∗^*p* < 0.05, ^∗∗^*p* < 0.01 vs. T2DM group.

According to the results of WB presented in Figures 9(a) and 9(b), Cyt-c and caspase-3 expression in the islet tissue of rats was significantly higher in the T2DM group than in the NC group, whereas the Bcl-2/Bax ratio was significantly decreased, indicating that apoptosis of islet cells was significantly increased in rats in the T2DM group. As the main islet cells, *β*-cells are reduced in number or dysfunctional, leading to insufficient INS secretion and elevated blood glucose levels, which are consistent with the aforementioned results for FBG [52]. Compared with the results in the T2DM group, Cyt-c and caspase-3 expression in the islet tissue of rats was significantly decreased after BFTL treatment, whereas the Bcl-2/Bax ratio was significantly increased, indicating that BFTL could improve T2DM by inhibiting apoptosis.

At normal blood glucose concentrations, glucose is not reduced by aldose reductase and only enters other metabolic pathways such as glycolysis. However, when blood glucose is elevated, a large amount of glucose enters the polyol pathway, sorbitol concentration increases, and NADPH is overconsumed [53, 54], whereas, NADPH has an important role in maintaining cellular redox, especially on the activity of glutathione peroxidase GPx and glutathione reductase (GRx). GRx uses NADPH as a cofactor and thus maintains GSH concentrations. In addition, the antioxidant activity of GPx decreases with the decrease in cellular GSH concentration [55]. When blood glucose is elevated, intracellular GSH content and GPx activity are significantly reduced, and excess O_2_ inhibits the function of the human antioxidant system, which in turn leads to a significant decrease in oxidative stress and the occurrence of antioxidant defenses against other biomolecules organism [56, 57].

Chronic persistent hyperglycemia can cause oxidative stimulation of pancreatic cells, and pancreatic beta cells are highly susceptible to oxidative attack by excess ROS due to their lower antioxidant enzyme content than other tissues. ROS can directly damage or oxidize DNA, proteins, and lipids, which can lead to beta cell dysfunction and death. In addition to damaging biomolecules, ROS can also activate a series of cellular stress-sensitive pathways, which in turn cause insulin resistance and inhibition of insulin secretion [58]. Current studies have shown that four signaling pathways are associated with oxidative stress in diabetes: the polyol pathway, the late glycation end product formation pathwayformation), the protein kinase C-diacylglycerol pathway, and the hexosamine pathwayl [59, 60]. In our study, we found that rats exhibited significantly reduced levels of TG, TC, MDA, ALT, and AST, and significantly increased levels of SOD and GSH-PX after treatment. So, BFTL exerted significant hypoglycemic effect on T2DM model rats.

## 5. Conclusions

**Figure 10:**
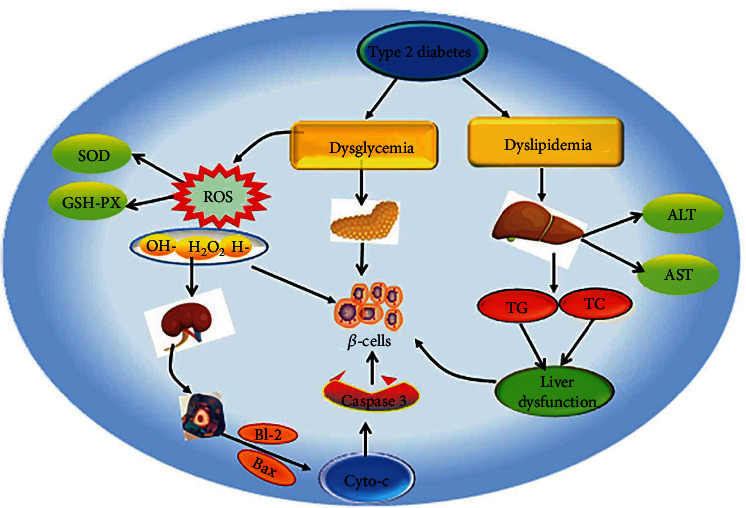
The hypoglycemic mechanism of BFTL on T2DM in rats.

The experimental results demonstrated that BFTL has an obvious hypoglycemic effect on T2DM in rats. In addition, the effect was more obvious in the high-dose BFTL groups than in the low- and middle-dose groups, suggesting a dose-dependent effect. Regarding its hypoglycemic mechanism, BFTL might induce pathological changes of the liver, kidneys, and pancreas in T2DM rats by improving their glucose and lipid metabolism, reducing oxidative stress, and improving the antioxidant capacity of the body, thus reversing INS-producing *β*-cell dysfunction and promoting INS secretion (Figure 10). Finally, the results of the present study indicated that the n-butanol fraction of *Torreya grandis* leaves showed several beneficial effects for the management of diabetes and thus it can be considered as a good candidate for further research in patients with diabetes. Meanwhile, it provided sufficient theoretical basis for future clinical research.

## Data Availability

All the data can be found in this paper.

## References

[1] Chen L., Lu X., El-Seedi H., Teng H. (2019). Recent advances in the development of sesquiterpenoids in the treatment of type 2 diabetes. *Trends in Food Science and Technology*.

[2] Liang W., Ye D. D. (2019). The potential of adipokines as biomarkers and therapeutic agents for vascular complications in type 2 diabetes mellitus. *Cytokine & Growth Factor Reviews*.

[3] Festa A., Heller S. R., Seaquist E., Duan R., Hadjiyianni I., Fu H. (2017). Association between mild and severe hypoglycemia in people with type 2 diabetes initiating insulin. *Journal of Diabetes and its Complications*.

[4] King B. C., Blom A. M. (2017). Non-traditional roles of complement in type 2 diabetes: metabolism, insulin secretion and homeostasis. *Molecular Immunology*.

[5] Pavlou D. I., Paschou S. A., Anagnostis P. (2018). Hypertension in patients with type 2 diabetes mellitus: targets and management. *Maturitas*.

[6] Li F., Abatan O. I., Kim H. (2006). Taurine reverses neurological and neurovascular deficits in Zucker diabetic fatty rats. *Neurobiology of Disease*.

[7] Wang N., Zhu F., Chen L., Chen K. (2018). Proteomics, metabolomics and metagenomics for type 2 diabetes and its complications. *Life Sciences*.

[8] Oza M. J., Kulkarni Y. A. (2018). Biochanin a improves insulin sensitivity and controls hyperglycemia in type 2 diabetes. *Biomedicine & Pharmacotherapy*.

[9] Delgado-León T. G., Sálas-Pacheco J. M., Vazquez-Alaniz F. (2018). Apoptosis in pancreatic *β*-cells is induced by arsenic, atorvastatin in Wistar rats with type 2 diabetes mellitus. *Journal of Trace Elements in Medicine and Biology*.

[10] Tatsuo T. (2016). Apoptosis in pancreatic *β*-islet cells in type 2 diabetes. *Bosnian journal of basic medical sciences*.

[11] Orchard T. J., Olson J. C., Erbey J. R. (2003). Insulin resistance-related factors, but not glycemia, predict coronary artery disease in type 1 diabetes. *Diabetes Care*.

[12] Shoorei H., Khaki A., Khaki A. A., Hemmati A. A., Moghimian M., Shokoohi M. (2019). The ameliorative effect of carvacrol on oxidative stress and germ cell apoptosis in testicular tissue of adult diabetic rats. *Biomedicine & Pharmacotherapy*.

[13] Shoorei H., Khaki A., Shokoohi M. (2020). Evaluation of carvacrol on pituitary and sexual hormones and their receptors in the testicle of male diabetic rats. *Human and Experimental Toxicology*.

[14] Abtahi-Eivari S. H., Shokoohi M., Ghorbani M. (2021). Effects of hydroalcoholic extracts of cloves (Syzygium aromaticum) on the serum biomarkers, antioxidant status, and histopathological changes of kidneys in diabetic rats. *Crescent Journal of Medical and Biological Sciences*.

[15] Abtahi-Evari S.-H., Shokoohi M., Abbasi A., Rajabzade A., Shoorei H., Kalarestaghi H. (2017). Protective effect of Galega officinalis extract on streptozotocin-induced kidney damage and biochemical factor in diabetic rats. *Crescent Journal of Medical and Biological Sciences*.

[16] Ni Q., Gao Q., Yu W., Liu X., Xu G., Zhang Y. (2015). Supercritical carbon dioxide extraction of oils from two _Torreya grandis_ varieties seeds and their physicochemical and antioxidant properties. *LWT- Food Science and Technology*.

[17] Niu L., Bao J., Mo J., Zhang Y. (2010). Chemical composition and mosquito Aedes aegyptirepellent activity of essential oil extracted from the aril ofTorreya grandis. *Journal of Essential Oil Bearing Plants*.

[18] Chen R. T., Zhang Y. H., Fang S. D. (1997). Inhibitors of human DNA polymerase isolated from Jack Torreya (Torreya jackii). *Chinese Traditional and Herbal Drugs*.

[19] Chen Z. D., Zheng H. C., Tian M. L., Zhang H., Zhu Z. M. (1998). Constituents analysis and antibacterial and antifungous actions of the essential oil in leaves of Torreya jackii Chun. *Academic Journal of Second Military Medical University*.

[20] Saeed M. K., Deng Y., Dai R., Li W., Yu Y., Iqbal Z. (2010). Appraisal of antinociceptive and anti-inflammatory potential of extract and fractions from the leaves of *Torreya grandis* Fort Ex. Lindl. *Lindl. J Ethnopharmacol*.

[21] Gurlo T., Ryazantsev S., Huang C. (2010). Evidence for proteotoxicity in *β* cells in type 2 diabetes: toxic islet amyloid polypeptide oligomers form intracellularly in the secretory pathway. *The American Journal of Pathology*.

[22] Olaniru O. E., Persaud S. J. (2018). Identifying novel therapeutic targets for diabetes through improved understanding of islet adhesion receptors. *Current Opinion in Pharmacology*.

[23] Akuodor G. C., Udia P. M., Bassey A., Chilaka K. C., Okezie O. A. (2014). Antihyperglycemic and antihyperlipidemic properties of aqueous root extract of *Icacina senegalensis* in alloxan induced diabetic rats. *Journal of Acute Disease*.

[24] Li J., Yuan K., Shang S., Guo Y. (2017). A safer hypoglycemic agent for type 2 diabetes--Berberine organic acid salt. *Journal of Functional Foods*.

[25] Cheng H. S., Ton S. H., Phang S. C., Tan J. B., Abdul K. K. (2017). Increased susceptibility of post-weaning rats on high-fat diet to metabolic syndrome. *Journal of Advanced Research*.

[26] Xu T., Ge Y., Du H. (2021). Berberis kansuensis extract alleviates type 2 diabetes in rats by regulating gut microbiota composition. *Journal of Ethnopharmacology*.

[27] Li B., Fu L., Kojima R., Yamamoto A., Ueno T., Matsui T. (2021). Theaflavins prevent the onset of diabetes through ameliorating glucose tolerance mediated by promoted incretin secretion in spontaneous diabetic Torii rats. *Journal of Functional Foods*.

[28] Heidarianpour A., Mohammadi F., Keshvari M., Mirazi N. (2021). Ameliorative effects of endurance training and *Matricaria chamomilla flowers* hydroethanolic extract on cognitive deficit in type 2 diabetes rats. *Biochemical Pharmacology*.

[29] Chen Y., Chen L., Yang T. (2021). Silymarin nanoliposomes attenuate renal injury on diabetic nephropathy rats *via* co-suppressing TGF- *β*/Smad and JAK2/STAT3/SOCS1 pathway. *Life Sciences*.

[30] Lu H., Sun J., Xu Y., Xie D., Sun L., Shu X. (2007). Association between the leptin receptor gene polymorphism and lipoprotein profile in Chinese type 2 diabetes. *Diabetes and Metabolic Syndrome: Clinical Research and Reviews*.

[31] Massaeli H., Pierce G. N. (1995). Involvement of lipoproteins, free radicals, and calcium in cardiovascular disease processes. *Cardiovascular Research*.

[32] Gu L., Ma Y., Gu M. (2015). Spexin peptide is expressed in human endocrine and epithelial tissues and reduced after glucose load in type 2 diabetes. *Peptides*.

[33] Jacobson T. A. M., Miller M. M., Schaefer E. J. M. (2007). Hypertriglyceridemia and cardiovascular risk reduction. *Clinical Therapeutics*.

[34] Guo L., Shi M., Zhang L. (2011). Galanin antagonist increases insulin resistance by reducing glucose transporter 4 effect in adipocytes of rats. *General and Comparative Endocrinology*.

[35] Norouzirad R., Gholami H., Ghanbari M. (2019). Dietary inorganic nitrate attenuates hyperoxia-induced oxidative stress in obese type 2 diabetic male rats. *Life Sciences*.

[36] Mostad I. L., Bjerve K. S., Basu S., Sutton P., Frayn K. N., Grill V. (2009). Addition of *n* -3 fatty acids to a 4-hour lipid infusion does not affect insulin sensitivity, insulin secretion, or markers of oxidative stress in subjects with type 2 diabetes mellitus. *Meta*.

[37] Chio I. I. C., Tuveson D. A. (2017). ROS in cancer: the burning question. *Trends in Molecular Medicine*.

[38] Zhu D., Wang H., Zhang J. (2015). Irisin improves endothelial function in type 2 diabetes through reducing oxidative/nitrative stresses. *Journal of Molecular and Cellular Cardiology*.

[39] Cheng F. R., Cui H. X., Fang J. L., Yuan K., Guo Y. (2019). Ameliorative effect and mechanism of the purified anthraquinone-glycoside preparation from rheum Palmatum L. on type 2 diabetes mellitus. *Molecules*.

[40] Wan H., Yang H., Xu G., Huang Y. (2020). Silkworm extract ameliorates type 2 diabetes mellitus and protects pancreatic *β* -cell functions in rats. *Digital Chinese Medicine*.

[41] Lo L., McLennan S. V., Williams P. F. (2011). Diabetes is a progression factor for hepatic fibrosis in a high fat fed mouse obesity model of non-alcoholic steatohepatitis. *Journal of Hepatology*.

[42] Monami M., Bardini G., Lamanna C. (2008). Liver enzymes and risk of diabetes and cardiovascular disease: results of the Firenze Bagno a Ripoli (FIBAR) study. *Meta*.

[43] Li Y., Wang J., Han X. (2019). Serum alanine transaminase levels predict type 2 diabetes risk among a middle- aged and elderly Chinese population. *Annals of Hepatology*.

[44] Herath H. M. M., Kodikara I., Weerarathna T. P., Liyanage G. (2019). Prevalence and associations of non-alcoholic fatty liver disease (NAFLD) in Sri Lankan patients with type 2 diabetes: a single center study. *Diabetes & Metabolic Syndrome: Clinical Research & Reviews*.

[45] Petersen K. F., Shulman G. L. (2006). New insights into the pathogenesis of insulin resistance in humans using magnetic resonance spectroscopy. *Obesity*.

[46] Deng T., Zhang Y., Wu Y. (2018). Dibutyl phthalate exposure aggravates type 2 diabetes by disrupting the insulin-mediated PI3K/AKT signaling pathway. *Toxicology Letters*.

[47] Jones D. A., Prior S. L., Barry J. D., Caplin S., Baxter J. N., Stephens J. W. (2014). Changes in markers of oxidative stress and DNA damage in human visceral adipose tissue from subjects with obesity and type 2 diabetes. *Diabetes Research and Clinical Practice*.

[48] Vairaktaris E., Kalokerinos G., Goutzanis L. (2008). Diabetes enhances cell proliferation but not Bax/Bcl-2-mediated apoptosis during oral oncogenesis. *International Journal of Oral and Maxillofacial Surgery*.

[49] Wu Y., Reece A., Zhong J. (2016). Type 2 diabetes mellitus induces congenital heart defects in murine embryos by increasing oxidative stress, endoplasmic reticulum stress, and apoptosis. *American Journal of Obstetrics and Gynecology*.

[50] Karunakaran U., Park S. J., Jun D. Y. (2015). Non-receptor tyrosine kinase inhibitors enhances *β*-cell survival by suppressing the PKC*δ* signal transduction pathway in streptozotocin - induced *β*-cell apoptosis. *Cellular Signalling*.

[51] Wing R. R., Rosen R. C., Fava J. L. (2010). Effects of weight loss intervention on erectile function in older men with type 2 diabetes in the look ahead trial. *The Journal of Sexual Medicine*.

[52] Yuan K., Yu L. (2005). Development and application of reduced pressure concentration device. *Chinese Journal of Analytical Chemistry*.

[53] Srivastava S. K., Ramana K. V., Bhatnagar A. (2005). Role of aldose reductase and oxidative damage in diabetes and the consequent potential for therapeutic options. *Endocrine Reviews*.

[54] Gleissner C. A., Sanders J. M., Nadler J., Ley K. (2008). Upregulation of aldose reductase during foam cell formation as possible link among diabetes. hyperlipidemia. And atherosclerosis. *Arteriosclerosis, Thrombosis, and Vascular Biology*.

[55] Acharya A., Das I., Chandhok D., Saha T. (2010). Redox regulation in cancer: a double-edged sword with therapeutic potential. *Oxidative Medicine and Cellular Longevity*.

[56] Styskal J., Van Remmen H., Richardson A., Salmon A. B. (2012). Oxidative stress and diabetes: what can we learn about insulin resistance from antioxidant mutant mouse models?. *Free Radical Biology & Medicine*.

[57] Makino A., Scott B. T., Dillmann W. H. (2010). Mitochondrial fragmentation and superoxide anion production in coronary endothelial cells from a mouse model of type 1 diabetes. *Diabetologia*.

[58] Einarson T. R., Acs A., Ludwig C., Panton U. H. (2018). Prevalence of cardiovascular disease in type 2 diabetes: a systematic literature review of scientific evidence from across the world in 2007-2017. *Cardiovascular Diabetology*.

[59] Brownlee M. (2001). Biochemistry and molecular cell biology of diabetic complications. *Nature*.

[60] Evans J. L., Goldfine L. D., Maddux B. A., Grodsky G. M. (2003). Are oxidative stress-activated signaling pathways mediators of insulin resistance and beta-cell dysfunction?. *Diabetes*.

